# Supplementary material for: Accelerating process development for 3D printing of new metal alloys
Source: Nat Commun. 2024 Jan 17;15:582. doi: 10.1038/s41467-024-44783-5 (PMC10794417; doi:10.1038/s41467-024-44783-5)
Supplement: Supplementary file 1 — Supplementary Information [file 41467_2024_44783_MOESM1_ESM.pdf]

Supplementary Information for

Accelerating Process Development for 3D Printing of New Metal Alloys

David Guirguis[1,2], Conrad Tucker[1,2,3], Jack Beuth[1,2]

[1] Next Manufacturing Center, Carnegie Mellon University
[2] Mechanical Engineering Department, Carnegie Mellon University
[3] Machine Learning Department, Carnegie Mellon University

This PDF file includes the following sections:

Supplementary Method
Supplementary Tables S1 to S10

Supplementary Method

To assess the performance of the method, comparisons are carried out with other state-of-the-art models. The configurations of the models and training details are as follows:

ViViT-B [1]: The factorized encoder model is used. In this model, the spatial and temporal encoders are separate. The spatial encoder models spatial interactions per temporal index, whereas the temporal encoder models the interactions along the temporal dimension. The “Base” architecture proposed in [1] is used. The tubelet size (i.e., size of each spatial-temporal input tube) is 16 x 2. The number of spatial transformer layers is 12, the number of temporal layers is 4, and the number of heads is 12. Data augmentation steps of random horizontal flipping and random cropping are used. Stochastic layer dropout [2] is used with a rate of 0.1. The model weights are initialized from a pretrained model on ImageNet-21K [3]. The optimization algorithm Adadelta [4] is used for training with an initial learning rate of 0.0008, a batch size of 8, and 50 total epochs. Exponential learning rate decay is used with a multiplicative factor of 0.95.

TimeSformer [5]: The divided space-time attention model is used. The transformer has 12 heads, and the number of layers is 12. The hidden dimension is 768. The size of the spatial-temporal input tube is 16 x 2. The model weights are initialized from a pretrained model on Kinetics-400 [6]. The same training setup as that used for ViViT-B is applied to train the TimeSformer model.

ResNet152 [7]: A deep convolutional neural network model with 152 layers that uses residual learning is used. The model weights are initialized from a pretrained model on ImageNet-21K [3]. Data augmentation steps of random horizontal flipping and random cropping are used. The Adam optimization algorithm [8] is used for training with a learning rate of 0.0001 and a batch size of 32. For efficient training, the weights of the top layer are first optimized, followed by the weights of the whole model. Early stopping is applied to prevent overfitting.

VGG16 [9]: A deep convolutional neural network model with small convolution filters of (3x3) and 16 layers is used. The Adam optimization algorithm [8] is used for training with a learning rate of 0.0001 and a batch size of 128. Early stopping is applied to prevent overfitting.

MoViNet-A3 [10]: A 3D convolutional neural network model for video recognition is used. The same model configuration that was used [10] is used in this study. The weights are initialized by a model trained on Kinetics 600 [11]. The Adam optimization algorithm [8] is used for training with a learning rate of 0.0001 and a batch size of 16. Early stopping is applied to prevent overfitting.

Supplementary Tables

Table S1.

Results of defect and processing regime prediction by training on IN718 and testing on SS316L

	Precision	Recall	F1-score	Samples
Desirable	0.81	0.95	0.88	166
Keyholing	0.98	0.94	0.96	441
Balling	0.60	0.75	0.67	142
Lack-of-fusion	0.94	0.79	0.86	291

Table S2.

Results of defect and processing regime prediction by training on IN718 and testing on Ti-6Al-4V

	Precision	Recall	F1-score	Samples
Desirable	0.70	0.92	0.79	170
Keyholing	1.00	0.84	0.91	795
Balling	0.66	0.99	0.79	176
Lack-of-fusion	0.81	0.77	0.79	309

Table S3.

Results of defect and processing regime prediction by training on Ti-6Al-4V and testing on IN718

	Precision	Recall	F1-score	Samples
Desirable	0.73	0.97	0.83	429
Keyholing	1.00	0.54	0.71	334
Balling	0.94	1.00	0.97	213
Lack-of-fusion	1.00	1.00	1.00	568

Table S4.

Results of defect and processing regime prediction by training on Ti-6Al-4V and testing on SS316L

	Precision	Recall	F1-score	Samples
Desirable	0.52	0.88	0.65	170
Keyholing	1.00	0.80	0.89	795
Balling	0.79	0.89	0.83	176
Lack-of-fusion	0.83	0.88	0.85	309

Table S5.

Comparison of the top-1 accuracy (%) results obtained by pretrained ViViT-B with model variants that include spatiotemporal attention, a factorized encoder and factorized self-attention [1]

Training on	Testing on	Spatiotemporal attention	Factorized encoder	Factorized self-attention
Ti-6Al-4V	SS316L	89.20	89.50	88.10
	IN718	94.90	95.60	95.10
SS316L	Ti-6Al-4V	91.40	89.70	91.10
	IN718	96.40	96.00	98.00
IN718	Ti-6Al-4V	95.80	92.70	94.20
	SS316L	89.40	92.30	88.80

Table S6.

Comparison of the top-1 accuracy (%) results obtained by pretrained ViViT-B model with and without data augmentation (random horizontal flipping and random cropping) and stochastic layer dropout (probability of 0.1)

Training on	Testing on	VIVIT-B (w/regularization)	VIVIT-B (w/o regularization)
Ti-6Al-4V	SS316L	90.24	85.62
	IN718	90.22	88.52
SS316L	Ti-6Al-4V	94.36	87.50
	IN718	94.48	96.54
IN718	Ti-6Al-4V	98.04	89.10
	SS316L	92.48	93.92

Table S7.

Chemical composition of the IN718 alloy (in weight %) according to the certification provided by the manufacturer; the specifications follow the ASTM B670 standard

**Al**	**B**	**C**	**Cb**	**Cb+Ta**	**Ca**	**Cr**	**Cu**	**Fe**	**Mn**	**Mo**	**Ni**	**P**	**S**	**Si**	**Ta**	**Ti**
0.55	0.003	0.04	5.03	5.04	0.34	18.36	0.06	18.96	0.23	3.08	52.57	0.008	<0.002	0.09	<0.05	1.02

Table S8.

Chemical composition of the SS316L alloy (in weight %) according to the certification provided by the manufacturer; the specifications are in compliance with the standards ASTM A751/20

**C**	**Co**	**Cr**	**Cu**	**Mn**	**Mo**	**N**	**Ni**	**P**	**Si**	**S**	**Fe**
0.01	0.2755	16.5675	0.4315	1.1945	2.0645	0.0565	10.03	0.0295	0.2505	0.001	Balance

Table S9.

Chemical composition of the Ti-6Al-4V alloy (in weight %) according to the certification provided by the manufacturer; the specifications are in compliance with the standards ASTM B265

	Fe	Al	C	V	N	Ti	O	H	Y
Min.	0.19	6.33	0.023	3.96	0.007		-	-	-
Max.	0.19	6.37	0.025	3.98	0.009	Balance	0.19	0.000038	0.0004

Table S10.

Chemical composition of the Ti-6Al-4V powder (EOS Titanium Ti64 Grade 23) in weight % according to the certification provided by the manufacturer; the specifications are in compliance with the standards ASTM F136, ASTM F3001, and ASTM F3302. Particle size 20-80 μm

	Al	V	Fe	C	O	N	Y	H	Ti
Min.	5.5	3.5	-	-	-	-	-	-	
Max.	6.5	4.5	0.25	0.08	0.13	0.05	0.005	0.012	Balance

Supplementary References

1. Anurag, A. *et al.* ViViT: A Video Vision Transformer. *IEEE/CVF International Conference on Computer Vision (ICCV)*, Montreal, QC, Canada, 6816-6826 (2021).
2. Huang, G., Sun, Y., Liu, Z., Sedra, D. & Weinberger, K. Q. Deep Networks with Stochastic Depth. *Lecture Notes in Computer Science (including subseries Lecture Notes in Artificial Intelligence and Lecture Notes in Bioinformatics)* **9908 LNCS**, 646–661 (2016).
3. Deng, J. *et al.* ImageNet: A large-scale hierarchical image database. *IEEE Conference on Computer Vision and Pattern Recognition*, Miami, FL, USA, 248-255 (2009).
4. Zeiler, M. D. ADADELTA: An Adaptive Learning Rate Method. (2012).
5. Bertasius, G., Wang, H. & Torresani, L. Is Space-Time Attention All You Need for Video Understanding? *Proc Mach Learn Res* **139**, 813–824 (2021).
6. Carreira, J., Zisserman, A., Quo Vadis, Action Recognition? A New Model and the Kinetics Dataset. *Proceedings - 30th IEEE Conference on Computer Vision and Pattern Recognition (CVPR)*, Honolulu, HI, USA, 4724–4733 (2017).
7. He, K., Zhang, X., Ren, S. & Sun, J. Deep Residual Learning for Image Recognition. *Proceedings of the IEEE Computer Society Conference on Computer Vision and Pattern Recognition (CVPR)*, Las Vegas, NV, USA, 770–778 (2015).
8. Kingma, D. P. & Ba, J. L. Adam: A Method for Stochastic Optimization. *3rd International Conference on Learning Representations, ICLR 2015 - Conference Track Proceedings* (2014).
9. Simonyan, K. & Zisserman, A. Very Deep Convolutional Networks for Large-Scale Image Recognition. *3rd International Conference on Learning Representations, ICLR 2015 - Conference Track Proceedings* (2014).
10. Kondratyuk, D. *et al.* MoViNets: Mobile Video Networks for Efficient Video Recognition. *Proceedings of the IEEE Computer Society Conference on Computer Vision and Pattern Recognition*, 16015–16025 (2021).
11. Long, F. *et al.* Learning to Localize Actions from Moments. *Lecture Notes in Computer Science (including subseries Lecture Notes in Artificial Intelligence and Lecture Notes in Bioinformatics)* **12348 LNCS**, 137–154 (2020).
