# Supplementary material for: Accelerating process development for 3D printing of new metal alloys
Source: Nat Commun. 2024 Jan 17;15:582. doi: 10.1038/s41467-024-44783-5 (PMC10794417; doi:10.1038/s41467-024-44783-5)
Supplement: Supplementary file 3 — Description of Additional Supplementary Files [file 41467_2024_44783_MOESM3_ESM.pdf]

Description of Additional Supplementary Files

File Name: Supplementary Movie S1
Description: Melt pool captured at 54000 fps during printing of single tracks of Ti-6Al-4V alloy in the keyholing regime at 350 W and 600 mm/s.

File Name: Supplementary Movie S2
Description: Melt pool captured at 54000 fps during printing of single tracks of Ti-6Al-4V alloy in the balling regime at 450 W and 1400 mm/s.

File Name: Supplementary Movie S3
Description: Melt pool captured at 54000 fps during printing of single tracks of Ti-6Al-4V alloy in the desirable regime at 200 W and 750 mm/s.

File Name: Supplementary Movie S4
Description: Melt pool captured at 54000 fps during printing of single tracks of Ti-6Al-4V alloy in the lack-of-fusion regime at 170 W and 1800 mm/s
